# Anthelmintic Drugs as Emerging Immune Modulators in Cancer

**DOI:** 10.3390/ijms24076446

**Published:** 2023-03-29

**Authors:** Carmine Stolfi, Teresa Pacifico, Anderson Luiz-Ferreira, Giovanni Monteleone, Federica Laudisi

**Affiliations:** 1 Department of Systems Medicine, University of Rome “Tor Vergata”, 00133 Rome, Italy; 2 Inflammatory Bowel Disease Research Laboratory, Department of Biological Sciences, Institute of Biotechnology, Federal University of Catalão (UFCAT), Catalão 75705-220, GO, Brazil; 3 Gastroenterology Unit, Policlinico Universitario Tor Vergata, 00133 Rome, Italy

**Keywords:** drug repositioning, cancer immunotherapy, immune checkpoint inhibitors, immunogenic cell death, PD-1, PD-L1, STAT3, niclosamide, rafoxanide, Th17

## Abstract

Despite recent advances in treatment approaches, cancer is still one of the leading causes of death worldwide. Restoration of tumor immune surveillance represents a valid strategy to overcome the acquired resistance and cytotoxicity of conventional therapies in oncology and immunotherapeutic drugs, such as immune checkpoint inhibitors and immunogenic cell death inducers, and has substantially progressed the treatment of several malignancies and improved the clinical management of advanced disease. Unfortunately, because of tumor-intrinsic and/or -extrinsic mechanisms for escaping immune surveillance, only a fraction of patients clinically respond to and benefit from cancer immunotherapy. Accumulating evidence derived from studies of drug repositioning, that is, the strategy to identify new uses for approved or investigational drugs that are outside the scope of the original medical indication, has suggested that some anthelmintic drugs, in addition to their antineoplastic effects, exert important immunomodulatory actions on specific subsets of immune cell and related pathways. In this review, we report and discuss current knowledge on the impact of anthelmintic drugs on host immunity and their potential implication in cancer immunotherapy.

## 1. Introduction

Cancer is one of the leading causes of death and an important barrier to increasing life expectancy worldwide. Taking into account all countries in the world, in 2020, an estimated 19.3 million new cancer cases and almost 10.0 million cancer deaths occurred [1]. Female breast cancer has surpassed lung cancer as the most commonly diagnosed cancer, with an estimated 2.3 million new cases (11.7%), followed by lung (11.4%), colorectal (10.0%), prostate (7.3%), and stomach (5.6%) cancers. Lung cancer remains the leading cause of cancer death, with an estimated 1.8 million deaths (18%), followed by colorectal (9.4%), liver (8.3%), stomach (7.7%), and breast (6.9%) cancers [1]. In general, the burden of cancer incidence and mortality is rapidly growing worldwide. This reflects both aging and growth of the population, as well as changes in the prevalence and distribution of the main risk factors for cancer, several of which are associated with socioeconomic development [2].

Currently, solid cancer therapy is focused on surgical removal, although this option is usually only available at the early stages of cancer development [3]. In the later stages, when metastases are detected, chemotherapy is the preferred treatment. Unfortunately, a significant number of patients can experience resistance to cancer drugs, either at the beginning or during the course of treatment, which eventually results in therapy failure [4]. These issues require other therapeutic options and are forcing researchers to seek new chemicals.

However, the development of new therapeutics has become increasingly difficult for pharmaceutical companies. In fact, classical drug discovery requires target identification and validation, evaluation of compound efficacy and pharmacology in vitro and in vivo, and analysis of toxicology, specificity, and potential drug interactions. This means that the process of selling a new drug on the market is costly and time-consuming, taking around 10–15 years to complete [5]. The extent of these challenges is revealed in an overall failure rate in drug development of more than 96% (with a failure rate of 90% during clinical development), and in the increasingly reduced number of new therapeutics approved by drug regulatory authorities [5].

Given these drawbacks, alternative drug discovery approaches should be employed to make drug research and development less time-consuming and financially demanding.

## 2. Drug Repositioning: Anthelmintic Drugs and Cancer Therapy

One such approach is drug repositioning (also known as drug repurposing); that is, the strategy to identify new uses for approved or investigational drugs that are outside the scope of the original medical indication [6]. Drug repositioning has many advantages that essentially reduce the regulatory process for the commercialization to market of already approved drugs. The procedure considers previously obtained data on bioavailability and absorption, distribution, metabolism, excretion, and toxicity (ADMET) profiles, thus, making the early stages of development significantly faster and more affordable [7]. The ideal candidates for drug repositioning are agents that have passed phase III in the FDA, as this means that they are adequate in large populations and safe [8]. Therefore, clinical trials can proceed much faster because the in vitro and in vivo screening has already been completed and passed. Drug repositioning has been very successful and has now become of particular interest to pharmaceutical companies. From the identification of various bioinformatic and cheminformatic methodologies, drug repositioning has evolved into a very innovative, data-driven, cutting-edge approach, guided by computational exploration aimed at detecting the relationship between various types of biological entities, such as genes, proteins, diseases, and drugs [9]. In this regard, anthelmintic drugs represent a very attractive class of compounds to be repurposed in oncology. Indeed, many of them show important anticancer properties as well as low toxicity in mammalian cells. In particular, anthelmintic drugs can inhibit cell proliferation and invasion of malignant cells, induce apoptotic cell death, and target key oncogenic transduction pathways in cancer cells without significantly affecting normal cell viability, whether used alone or in combination with conventional anticancer therapies [10]. For example, compounds belonging to the class of benzimidazoles, such as mebendazole and albendazole, were found to exert cytostatic and cytotoxic effects on colorectal cancer (CRC) and hepatocellular carcinoma (HCC)-derived cell lines, as well as in vivo models [11,12,13,14,15,16]. Similarly, the halogenated salicylanilide compounds niclosamide, rafoxanide, and closantel have been reported to affect cancer cell proliferation and viability, to block the Wnt/β-catenin signaling pathway, and reduce tumor burden and liver metastasis in mouse models mimicking sporadic CRC [17,18,19,20,21]. Furthermore, niclosamide showed significant anticancer activity in pancreatic and esophageal cancer [22,23]. All these findings, together with similar observations regarding other anthelmintic compounds, make such drugs extremely attractive for a possible therapeutic application and prompted several researchers to test them in clinical trials to treat cancer patients [24,25,26,27].

## 3. Immunomodulatory Functions of Anthelmintics

Most solid malignancies comprise not only tumor cells but also fibroblasts, vascular endothelial cells, extracellular matrix, many types of immune cells that exert both immunosuppressive functions [e.g., tumor-associated macrophages, myeloid-derived suppressor cells (MDSCs), and regulatory T-cells] and tumor-fighting functions (e.g., cytotoxic CD8^+^ T-cells, CD4^+^ Th1, natural killer cells), as well as multiple extracellular soluble molecules (e.g., cytokines, growth factors, chemotactic factors). This complex and heterogeneous ecosystem, defined as the tumor microenvironment [28], greatly affects the course of the disease and the functional state of tumor-infiltrating immune cells has become, over the past decade, an important prognostic and predictive factor in the fate of cancer patients treated with conventional or targeted therapies [29,30].

The tumor microenvironment could be simply defined as “cold” (non-T-cell-inflamed) or “hot” (T-cell-inflamed), which is largely attributed to the levels of proinflammatory cytokines and T-cell infiltration [31]. These so-called hot tumors are characterized by T-cell infiltration and molecular signatures of immune activation, whereas cold tumors show striking features of T-cell absence or exclusion [31]. In general, hot tumors present higher response rates to immunotherapeutic drugs, such as those targeting programmed death protein 1 (PD-1) and its ligand programmed death-ligand 1 (PD-L1) [32], which mediate co-inhibitory signals to T-cell activation, resulting in the attenuation of the host immune response to tumor cells [33].

In this respect, it is important to underline that the potential antitumor effects of various anthelmintic drugs are not limited to the cytostatic and pro-apoptotic properties mentioned above, as several studies highlighted their ability to modulate host immunity. In particular, the activity of different immune cells and transcription factors [e.g., signal transducer and activator of transcription 3 (STAT3), nuclear factor kappa-light-chain-enhancer of activated B cells (NF-κB)], which are involved in the cancer-related immune response, can be influenced by these compounds [34,35]. Furthermore, recent reports described the efficacy of some anthelmintics in inducing immunogenic cell death (ICD) [21], a special form of apoptosis associated with endoplasmic reticulum (ER) stress and release of damage-associated molecular patterns (DAMPs) [36], or to synergize with PD-1/PD-L1 blockade immunotherapy [37].

Here, we review and discuss the experimental and clinical evidence on the ability of anthelmintic drugs to exert immunomodulatory properties to improve cancer treatment.

### 3.1. Effects on Immune Cells

Early observations in the 1990s indicated that some anthelmintic agents, such as ivermectin, have important immunomodulatory properties in vivo after subcutaneous treatment of CD1 mice [38]. However, such observations have not been considered for several years, and the concept of anthelmintic drugs as potential immunomodulators in cancer therapy has resumed only recently, with the addition of many other agents to this list (Table 1).

For instance, among the benzimidazole compounds, the molecules N-acyl-2-aminobenzimidazole-1 and -2 were able to block the interleukin-1 receptor-associated kinase (IRAK)-4 function, thus, affecting IL-1 signal transduction [39]. In vitro studies with bone-marrow-derived macrophages show that N-acyl-2-aminobenzimidazole-1 and -2 inhibit the toll-like receptor (TLR) downstream effectors IRAK1 and IRAK4, leading to a significant reduction in mitogen-activated protein (MAP) kinase phosphorylation, NF-κB nuclear translocation, and subsequent release of cytokines [40]. In a model of murine sterile inflammation, the compounds reduced splenocyte proliferation, the expression of markers associated with chronic inflammation (e.g., kininogen, kallikrein, and fibronectin), as well as the production of proinflammatory chemokines (e.g., CCL-2, CCL-5, CCL-17), without affecting macrophage activation [40]. Within the benzimidazole class, albendazole was also reported to exert important immunomodulatory properties in psoriasis [41], a dermatologic disease associated with an increased risk of developing cancer (i.e., colorectal, skin, gastric, and lung cancer) in selected subgroups of patients [53]. Data from a mouse model of Aldara-induced psoriasis show that local administration of albendazole on psoriatic plaques results in the arrest of keratinocyte proliferation and a lower frequency of infiltrating T lymphocytes and neutrophils, together with a reduced expression of pro-inflammatory molecules (e.g., IL-6, TNF-α, IL-1β, IL-17A, IL-36, CCL17, CXCL1, CXCL2, CXCL5) [41]. The findings of Larsson’s group show that mebendazole, another benzimidazole compound, induces a tumor-suppressive M1 phenotype in the primed/activated human leukemia monocytic THP-1 cell line, which significantly affects tumor cell growth once co-cultured with colorectal adenocarcinoma HT-29 cells [42]. Later, the same research group reported that mebendazole potentiated the immune stimulatory and anticancer effects of anti-CD3/IL2-activated peripheral blood mononuclear cells (PBMCs) against lung cancer cells by polarizing CD14^+^ monocytes toward a macrophage profile M1. [43]. Another important anthelmintic drug that can target the immune response, even in the context of cancer, is flubendazole. This compound has been reported to trigger an antitumor response by blocking the activity of the NF-κB pathway [44]. In detail, Tao et al. show that flubendazole inhibits the activation of IκBα kinases (IKK), resulting in decreased phosphorylation of the NF-κB p65 subunit in squamous esophageal carcinoma cells [44]. Flubendazole also interferes with STAT3 activity, as well as with the function/differentiation of T helper (Th)-17 cells and regulatory T-cells, thus, sustaining an antitumor response in triple-negative breast cancer (TNBC) [46], CRC [45], and non-small-cell lung cancer (NSCLC) [47]. In addition to its inhibitory effect on breast cancer cell proliferation, viability, and angiogenesis, the halogenated salicylanilide compound niclosamide was shown to reduce the frequency of infiltrated myeloid-derived suppressor cells (MDSCs), which are known to negatively affect antitumor immune responses [54], in the tumor niche [48]. Furthermore, niclosamide was reported to influence the activation of other immune cell subsets, such as dendritic cells (DCs), resulting in a reduced production of pro-inflammatory cytokines (i.e., TNF-α and IL-6) and chemokines (e.g., MIP1, MCP1) by bone-marrow-derived DCs in response to lipopolysaccharides (LPS) [49]. However, it should be noted that some findings indicate that niclosamide impairs the antigen-specific activation of T-cells by DCs due to the downregulation of costimulatory and major histocompatibility complex (MHC) molecules on the cell membrane, which are, instead, crucial to prime cytotoxic T-cell response against cancer [49]. Opposite effects were observed in DCs derived from human monocytes after treatment with levamisole, an imidazothiazole derivative. In fact, Chen and co-workers demonstrated that this anthelmintic agent could promote the expression of costimulatory and human leukocyte antigen (HLA)-DR molecules on the cell membrane of DCs, the release of IL-12p40 and IL-10 cytokines through TLR2 signaling, and the activation and differentiation of T-cells toward a Th1 phenotype [50]. Some years later, similar results were obtained from murine bone-marrow-derived DCs, where levamisole was shown to exert a positive effect on the expression of MHC and costimulatory molecules, the release of cytokines, and Th1 differentiation of CD4^+^ T-cells, both in vitro and in vivo [51]. It is noteworthy that in a 2009 study, levamisole was reported to efficiently boost TLR7/8 and interferon regulatory factor (IRF)-7 signaling, DC activation, and synergize with alum adjuvant to increase the immunogenicity of recombinant hepatitis B virus surface antigen (rHBsAg), thus, boosting the cell-mediated immune response against HBV infection [52]. Taken together, these results are consistent with the potential therapeutic use of levamisole in increasing the cytotoxic and immune response in cancer. 

### 3.2. Combination Antitumor Immunotherapy

**Figure 1:**
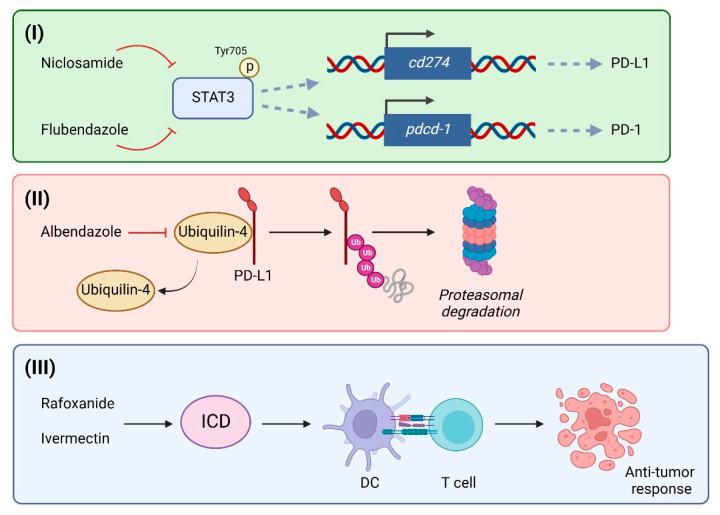
Some putative molecular mechanisms that underlie the ability of certain anthelmintic drugs to improve cancer immunotherapy. (**I**) Niclosamide and flubendazole suppress STAT3 phosphorylation/activation, thus, impairing the expression of PD-L1 and PD-1. (**II**) Albendazole negatively affects ubiquilin-4 expression/interaction with PD-L1, thus, promoting its degradation by the proteasome. (**III**) Rafoxanide and ivermectin promote bona fide immunogenic cell death in cancer cells and prime antitumor immune responses. Abbreviations: STAT- signal transducer and activator of transcription; PD-1: programmed cell death protein-1; PD-L1: programmed death-ligand 1; Ub: ubiquitin; ICD: immunogenic cell death; DC: dendritic cells. Created with Biorender.com.

Acquired resistance and high toxicity are among the main limitations of conventional cancer therapies [55]. In recent years, the use of immune checkpoint inhibitors in oncology, as the first or second line of treatment, has become a valuable alternative therapeutic approach to enhance the immune system of the host against various types of cancer and obtain a durable clinical response [56]. Today, the use of monoclonal antibodies anti-PD-1 (e.g., cemiplimab, nivolumab, pembrolizumab) and anti-PD-L1 (e.g., atezolizumab, avelumab, durvalumab) are quite common anticancer therapies [57]. However, clinical practice also showed us some limits of such an approach. First, immune checkpoint inhibitors appear to be particularly effective in hot tumors, such as metastatic melanoma, renal cancer carcinoma, and NSCLC, thus limiting the number of patients eligible for this treatment [32,58]. Furthermore, several immune-mediated adverse effects can arise quickly and affect the quality of life of patients so much that they must discontinue therapy [59,60]. Another not negligible problem is that a large group of patients (4–29%) experienced hyper-progressive disease, characterized by rapid tumor growth and spread of metastases, as well as decreased overall survival [61]. Finally, several patients, even if considered good candidates, do not respond to immune checkpoint inhibitors and develop primary or acquired resistance, highlighting the need to discover more effective alternatives. In this regard, the repositioning of anthelmintic drugs has recently emerged as an interesting approach to improve the clinical outcomes of immunotherapy (Figure 1).

In support of this view, studies highlight the ability of various anthelmintic drugs to enhance the efficacy of immune checkpoint blockade strategies. Luo and colleagues show that niclosamide decreases PD-L1 expression by affecting STAT3 phosphorylation and its binding to the PD-L1 promoter in NSCLC [37]. The results obtained in cultured cells and in vivo confirm that niclosamide combination therapy with anti-PD1/PD-L1 antibody delays tumor growth and successfully promotes cytotoxic antitumor immune responses [37]. More recently, interesting results were obtained using albendazole, which was found to target ubiquilin-4, a protein encoded by the *Ubqln4* gene, which interacts with and stabilizes PD-L1 [62]. The decrease in expression of the *Ubqln4* gene resulted in ubiquitin-mediated degradation of PD-L1 and increased activity of cytotoxic T lymphocytes in melanoma cells both in vitro and in vivo [62]. Similar results were obtained by testing other benzimidazole derivatives, such as flubendazole, which was able to target PD-1, but not PD-L1, in melanoma cells, and negatively affect tumor infiltration of MDSCs by inhibiting STAT3 phosphorylation [63]. Screening a large database of approved or investigational drugs helped identify several small molecules capable of inducing dimerization and sequestration of PD-L1. Among them, the anthelmintic drug pyrvinium was found to be the most relevant dimerizer of PD-L1 [64]. Even if experimental pieces of evidence on its antitumor efficacy in vitro and in vivo are still missing, these results pave the way for the development of additional structural analogs of pyrvinium, and support the idea that similar computational approaches could be employed to identify or repurpose approved drugs as potential PD-1/PD-L1 inhibitors. In addition to their ability to modulate PD-1/PD-L1 expression and, therefore, to improve the efficacy of immune checkpoint inhibitors, some anthelmintic agents also recently revealed their ability to make some types of tumors more “immunogenic” by promoting a particular form of apoptosis called immunogenic cell death (ICD), which allows the cancer cells to be recognized and targeted by the immune system [21,65]. ICD presents distinctive features compared to apoptotic or necrotic cell death, characterized by the exposure of calreticulin on the cell membrane, the release of soluble mediators, such as adenosine triphosphate (ATP) and the high-mobility group box-1 protein (HMGB1), generally triggered by ER stress, and the activation of DCs, which can easily prime the antitumor immune response [66,67]. In this context, Draganov et al. show that the anthelmintic agent ivermectin induces ICD in TNBC, a typical, non-immunogenic, “cold” tumor, characterized by cells that can suppress the immune response and prevent T-cells from attacking malignant cells, by activating the ATP/P2X4/P2X7 signaling axis, apoptotic and necrotic cell death, as well as mitochondrial damage, ER stress, ATP release, inflammasome activation, and autophagy [65]. Furthermore, recent data from the same group show that ivermectin improves the efficacy of anti-PD-1 therapy in a mouse model of breast cancer [68]. In detail, combination therapy was able to induce a strong antitumor immune response, characterized by increased tumor infiltration of CD4^+^ and CD8^+^ T-cells and a reduced frequency of MDSCs, resulting in increased tumor regression and prolonged survival in metastatic mice [68]. In line with these findings, we reported that rafoxanide triggered all the main hallmarks of ICD, that is, pre-mortem exposure of the calreticulin protein on the cell membrane, ER stress/EIF2α phosphorylation, as well as ATP/HMGB1 release, in cultured CRC cells [21]. To test our hypothesis that rafoxanide was an inducer of ICD in vivo, we employed the BALB/c-derived colon adenocarcinoma cell line CT26 in a vaccination setting. Pretreatment of immunocompetent BALB/c mice with rafoxanide-treated dying CT26 cells markedly suppressed the subsequent growth of CT26-derived tumors and increased tumor-free survival compared to sham mice, suggesting the establishment of a productive antitumor immune response [21]. Together, these results indicate that both ivermectin and rafoxanide are bona fide inducers of ICD that could be deployed to improve the clinical response of cancer patients to immunotherapy. It should be noted that a phase II clinical trial is underway to evaluate the best dose and tolerability of ivermectin in combination with pembrolizumab in patients with metastatic TNBC (ClinicalTrials.gov, identifier: NCT05318469). This trial aims to evaluate the ability of ivermectin to synergize with balstilimab, an anti-PD-1 monoclonal antibody, by characterizing the tumor immune profile and changes in the tumor microenvironment after treatment, as well as the ICD rate in tumor cells and progression-free survival, overall survival, duration of response, and clinical benefit rate of patients. 

Regarding rafoxanide, we recently showed that the drug acts as a selective TNF-related apoptosis-inducing ligand (TRAIL) sensitizer in vitro and in a syngeneic experimental model of CRC, by decreasing levels of c-FLIP and survivin [69], two key molecules conferring resistance to TRAIL [70]. Taken together, our data suggest that rafoxanide could potentially be used as an anticancer drug in combination approaches to overcome CRC cell resistance to TRAIL-based therapies.

Different studies assessed that ICD could be triggered by oxidative stress and mitochondrial dysfunction, and drugs able to trigger such mechanisms could be potentially used in combination with conventional cancer therapy and immunotherapy [71]. For example, ivermectin was found to promote mitochondrial dysfunction and oxidative stress in leukemia cells, and to synergize with cytarabine and daunorubicin to boost ROS production [72]. Similar effects have also been reported in chronic leukemia cells [73]. Furthermore, ivermectin was also found to affect mitochondrial respiration, membrane potential, and ATP levels, and to trigger oxidative stress in human glioblastoma cells (U87, T98 G, and HBMEC) [74]. Interesting results were also obtained in cervical cancer cells with the use of niclosamide, which could improve the response to paclitaxel treatment by inhibiting mitochondrial respiration, complex I activity, and ATP secretion, thus, leading to mitochondrial dysfunction and oxidative stress, and the consequential impairment of the mammalian target of the rapamycin (mTOR) signaling pathway [75].

## 4. Discussion

Restoration of tumor immune surveillance represents a valid strategy to overcome acquired resistance and cytotoxicity of conventional therapies in oncology. In particular, immunotherapeutic drugs, such as immune checkpoint inhibitors and immunogenic cell death inducers, have substantially advanced the treatment of several malignancies and improved the clinical management of advanced disease [76,77]. Unfortunately, only a fraction of patients clinically respond to and benefit from the above-mentioned therapies, due to tumor-intrinsic and/or -extrinsic mechanisms for escaping immune surveillance. Furthermore, some immunotherapy-related side effects (e.g., toxicity, unwanted immune-mediated reactions, hyper- and pseudo-progression of the disease) may jeopardize the quality of life and survival in certain patient populations. In recent years, experimental evidence revealed an unexpected role for some anthelmintic agents in modulating the host’s immune response in cancer treatment. For example, some anthelmintic drugs have been shown to subvert an immunosuppressive tumor microenvironment by boosting antigen presentation by dendritic cells and/or Th1-mediated/cytotoxic immune responses, as well as by impairing the infiltration of immunosuppressive cell subsets (i.e., MDSCs) into the tumor niche. All these findings led to major efforts to test the most promising compounds in combination with immune checkpoint inhibitors or ICD inducers to improve immunotherapy outcome.

However, despite all the enthusiasm and promising experimental and preclinical findings, the efficacy in clinical trials—as commonly occurs with drugs repurposed in other contexts—remains the main bottleneck for the successful repositioning of anthelmintic compounds as immune modulators in cancer treatment. To date, only a phase II clinical trial is planned to investigate the effects of ivermectin in combination with pembrolizumab in patients with TNBC (NCT05318469, no recruitment stage), whereas others aimed at assessing the antineoplastic effects of some anthelmintic drugs are either not updated (e.g., NCT04296851, NCT02519582, NCT03950518, NCT03940378), or were discontinued due to lack of effect (e.g., NCT03628079) (Table 2). 

These disappointing achievements may be, in part, due to the shortage of studies employing preclinical in vivo models and/or cancer-patient-derived cells/samples to assess the immunomodulatory properties of these agents. More robust data on these aspects would likely contribute to the selection of anthelmintic drugs with a greater potential for success in the clinic, although it is worth noting that all therapeutics have to deal with other issues related to the heterogeneity of primary tumors and metastases [78,79]. Furthermore, given that most studies do not have data on normal cells/tissues, future experimental work should consider the potential toxicity of repurposed drugs for non-cancer cells (e.g., immune cells, stromal cells, other non-transformed cells) after short-term and long-term administrations. Other possible pitfalls for the successful repositioning of anthelmintic drugs in oncology relate to their physicochemical properties and method of administration. In fact, anthelmintic drugs are administered orally and reach high concentrations in the gastrointestinal tract, but usually very low levels in circulation [80]. Thus, one of the advantages of drug repositioning (that is, knowledge of previous safety data) would not apply in the case of a systemic application. In addition, to achieve sufficient effectiveness of repurposed anthelmintics for cancer therapy, treatment may be required at higher doses and/or for longer periods compared to conventional indication, thereby resulting in unexpected side effects. To solve these problems, strategies are being tested to increase the solubility and decomposition rate of anthelmintic drugs (e.g., nanocrystals and lipid-based formulas) [81] to improve their oral absorption and achieve therapeutic blood levels.

## 5. Conclusions

Taken together, the studies described and discussed in this review suggest a promising role for some anthelmintic drugs as adjuvant agents in cancer immunotherapy. However, they have not been properly or sufficiently evaluated in clinical trials and some drawbacks need to be addressed and overcome to increase the likelihood of success in clinical practice.

## Figures and Tables

**Table 1:** Immunomodulatory properties of anthelmintic drugs.

Drug	Cell Type	Immunomodulatory Effect	Reference
1-(2-(4-Morpholinyl)ethyl)-2-(3-nitrobenzoylamino) benzimidazole (Benzimidazole derivaties)	(In silico)	< IL-1 signaling.	[39]
	BMDMs	< TLR signaling, MAPK phosphorylation, and nuclear translocation of NF-ĸB. < Pro-inflammatory cytokine/chemokine release.	[40]
	Splenocytes/serum	< Splenocyte proliferation. < Serum levels of pro-inflammatory cytokines.	[27]
Albendazole	Keratinocytes	< Infiltration of T-cells and neutrophils in psoriatic plaques. < Expression of pro-inflammatory cytokines and chemokines.	[41]
Mebendazole	THP-1	M1 polarization of THP1 and arrest of HT-29 cell proliferation.	[42]
	PBMCs	M1 polarization of CD14^+^ monocytes/Mϕ in αCD3/IL-2 stimulated PBMCs, release of pro-inflammatory cytokines, and induction of tumor cell apoptosis once co-cultured with A549NucLightRed cells.	[43]
Flubendazole	ESCC	< NF-ĸB activity by inhibition of IĸBα kinase function and phosphorylation of the p65 subunit.	[44]
	TNBC/CRC/NSCLC	< STAT3 activity.	[45,46,47]
Niclosamide	Breast cancer	< MDSC tumor infiltration.	[48]
	DCs/T cells	< Production of pro-inflammatory cytokines and chemokines in response to LPS. < T-cell priming.	[49]
Levamisole	Mo-DCs	> HLA-DR expression, IL-10/IL-12p40 release, and Th1 cell differentiation.	[50]
	BMDCs	> MHC expression, IL-12p70, TNF-α and IL-1β release, and Th1 cell differentiation.	[51]
	DCs/T cells	> TLR7/8 and IRF7 signaling, DCs and CTL activation, and IFN-γ production	[52]

Abbreviations: IL-: interleukin-; BMDM: bone-marrow-derived macrophages; TLR: toll-like receptor; MAPK: mitogen-activated protein kinase; NF-ĸB: nuclear factor kappa-light-chain-enhancer of activated B cells; Mϕ: macrophages; STAT: signal transducer and activator of transcription; MDSC: myeloid-derived suppressor cell; LPS: lipopolysaccharides; DC: dendritic cell; HLA-DR: human leukocyte antigen-DR isotype; Th1: T-helper-1; MHC: major histocompatibility complex; TNF: tumor necrosis factor; IRF-: interferon regulatory factor; CTL: cytotoxic T lymphocytes; IFN: interferon. STAT: signal transducer and activator of transcription; PD-1: programmed cell death protein-1; PD-L1: programmed death-ligand 1; Ub: ubiquitin; ICD: immunogenic cell death; DC: dendritic cells.

**Table 2:** Current clinical trials employing anthelmintic drugs to treat cancer.

Drug	Pathology	Official Title of the Study	Phase	Status	Identifier
Ivermectin	Metastatic TNBC	A Phase I/II Study Evaluating the Safety and Efficacy of Ivermectin in Combination with Balstilimab in Patients with Metastatic Triple-Negative Breast Cancer with Expansion Cohort in PD-L1 Negative TNBC	I/II	Not yet recruiting	NCT05318469
Niclosamide	FAP	The Chemopreventive Effect of Niclosamide in Patients with Familial Adenomatous Polyposis: Double-Blinded Randomized Controlled Study	II	Last update 5 March 2020	NCT04296851
Niclosamide	CRC	Phase II Trial to Investigate the Safety and Efficacy of Orally Applied Niclosamide in Patients with Metachronous or Synchronous Metastases of a Colorectal Cancer Progressing After Therapy	II	Last update 12 September 2018	NCT02519582
Levamisole	Advanced HCC	Multicenter, Randomized, Open, Parallel, Prospective, Exploratory Clinical Study of Arginine Hydrochloride and Levamisole in the Treatment of Advanced HCC	III	Recruiting	NCT03950518
Levamisole	Advanced ICC	The Efficacy of Levamisole Hcl in Advanced Intrahepatic Cholangiocarcinoma. A Multicenter, Open, Randomized, Prospective Study	III	Last update 7 May 2019	NCT03940378
Mebendazole	Advanced GI cancer of cancer of unknown origin	A Phase 2a TDM-guided Clinical Study on the Safety and Efficacy of Mebendazole in Patients with Advanced Gastrointestinal Cancer or Cancer of Unknown Origin	II	Terminated (lack of effect)	NCT03628079

Abbreviations: TNBC: triple-negative breast cancer; FAP: familial adenomatous polyposis, CRC: colorectal cancer, HCC: hepatocellular carcinoma, ICC: intrahepatic cholangiocarcinoma; GI: gastrointestinal.

## Data Availability

No new data were created or analyzed in this study. Data sharing is not applicable to this article.
